# Ultrafast dynamics of quasiparticles and coherent acoustic phonons in slightly underdoped (BaK)Fe_2_As_2_

**DOI:** 10.1038/srep25962

**Published:** 2016-05-16

**Authors:** Kung-Hsuan Lin, Kuan-Jen Wang, Chung-Chieh Chang, Yu-Chieh Wen, Bing Lv, Ching-Wu Chu, Maw-Kuen Wu

**Affiliations:** 1 Institute of Physics, Academia Sinica, Taipei 11529, Taiwan; 2 Institute of Photonics Technologies, National Tsing Hua University, Hsinchu 30013, Taiwan; 3 Texas Center for Superconductivity, University of Houston, Houston, TX 77004, USA; 4 Department of Physics, The University of Texas at Dallas, Richardson, TX 75080, USA

## Abstract

We have utilized ultrafast optical spectroscopy to study carrier dynamics in slightly underdoped (BaK)Fe_2_As_2_ crystals without magnetic transition. The photoelastic signals due to coherent acoustic phonons have been quantitatively investigated. According to our temperature-dependent results, we found that the relaxation component of superconducting quasiparticles persisted from the superconducting state up to at least 70 K in the normal state. Our findings suggest that the pseudogaplike feature in the normal state is possibly the precursor of superconductivity. We also highlight that the pseudogap feature of K-doped BaFe_2_As_2_ is different from that of other iron-based superconductors, including Co-doped or P-doped BaFe_2_As_2_.

Iron-based high-temperature superconductors have attracted great attention in recent years^1^. Among the more than six families based on their respective compositions of compounds, of which the 1111 (such as SmFeAsO), 122 (such as BaFe_2_As_2_), 111 (such as LiFeAs) and 11 (such as FeSe) systems have been widely studied. Compounds of the 122 system are the easiest among the iron-based superconductors to synthesize in the form of large single crystals, and most publications have been devoted to 122 compounds^2^. Because superconductor-based devices require high quality crystals/films, the 122 system has also been employed most often among the iron-based compounds to demonstrate devices such as Josephson junctions^3^,^4^,^5^,^6^ and superconducting wires/tapes for high current applications^7^,^8^,^9^,^10^,^11^,^12^,^13^,^14^,^15^,^16^. Although the unconventional high-temperature superconductors (including copper-based and iron-based superconductors) have been applied for devices, the fundamental questions such as the forming mechanisms of Cooper pairs remain unclear^17^. K-doped BaFe_2_As_2_ was the first compound in the 122 system found to exhibit superconductivity^18^. It should be noted that its parent compound, BaFe_2_As_2,_ has a structural and magnetic phase transition^19^, but Ba_1−x_K_x_Fe_2_As_2_ is only superconducting (SC) for a range of x with maximum T_c_ at x = 0.4^20^. Ba_1−x_K_x_Fe_2_As_2_ does not have a structural and magnetic phase transition when x is close to the optimal value. The phase diagram and planar geometry of K-doped BaFe_2_As_2_ are similar to those of cuprate superconductors, leading to the intriguing question of whether K-doped BaFe_2_As_2_ and cuprate superconductors have the same mechanism of superconductivity.

One of the general features in cuprate superconductors is the presence of a pseudogap above T_c_^21^. There are generally two energy scales for pseudogaps in cuprate superconductors and the low-energy pseudogap is believed to be the precursor of the SC gap^21^. However, there are contradictory results about the existence of pseudogap in optimally doped (BaK)Fe_2_As_2_ (BKFA) from angle-resolved photoemission spectroscopy (ARPES) measurements^22^,^23^. Although ARPES and scanning tunneling microscopy are capable of directly resolving band structures, they are surface-sensitive techniques. In contrast, ultrafast optical spectroscopy^24^,^25^,^26^,^27^,^28^,^29^,^30^ is not surface-sensitive, and is a convenient tool to study the bulk property of materials. By observing the temperature-dependent quasiparticle-relaxation, a pseudogaplike feature has been observed in FeSe^25^, LiFeAs^26^, Ba(Fe,Co)_2_As_2_ 27, Ca(Fe,Co)_2_As_2_ 28, SmFeAsO_0.8_F_0.2_ 29, and underdoped BKFA^30^.

In this article, we have utilized ultrafast optical spectroscopy to study carrier dynamics in slightly underdoped BKFA single crystals without magnetic transition. The signals of coherent acoustic phonons were quantitatively studied. We also found that the quasiparticle relaxation associated with the SC gap has temperature dependence that appears in the SC state and persists at least up to 70 K in the normal state. Our result supports the argument that the pseudogaplike feature in the normal state is possibly the precursor of superconductivity^30^,^31^. However, in most other iron-based superconductors^26^,^28^,^32^,^33^,^34^,^35^,^36^,^37^,^38^, the pseudogap is not associated with the SC gap and both of them coexist in the SC state. In addition, we suggest that the contradictory reports^22^,^23^ resulted from slightly different concentrations of K near optimally doped BKFA. The pseudogaplike feature could be found in slightly underdoped BKFA but was not found in optimally doped BKFA.

## Results and Discussions

**Figure 1:**
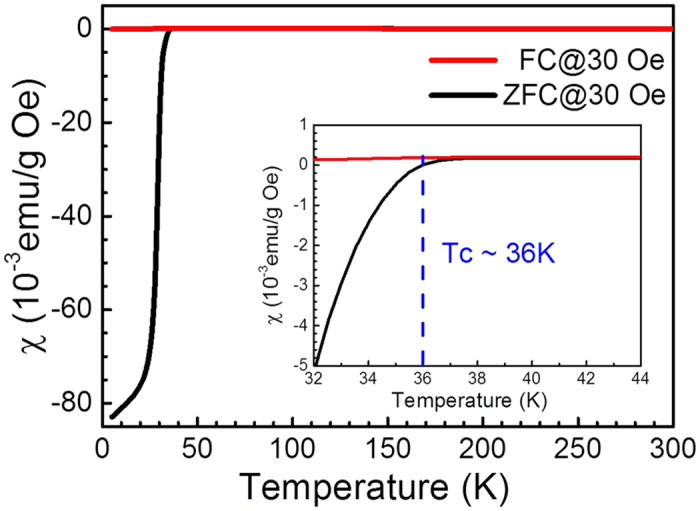
The temperature-dependent dc magnetic susceptibility of BKFA measured in field cooling (FC) and zero field cooling (ZFC) modes. The inset shows the magnetic susceptibility around the superconducting transition temperature.

**Figure 2:**
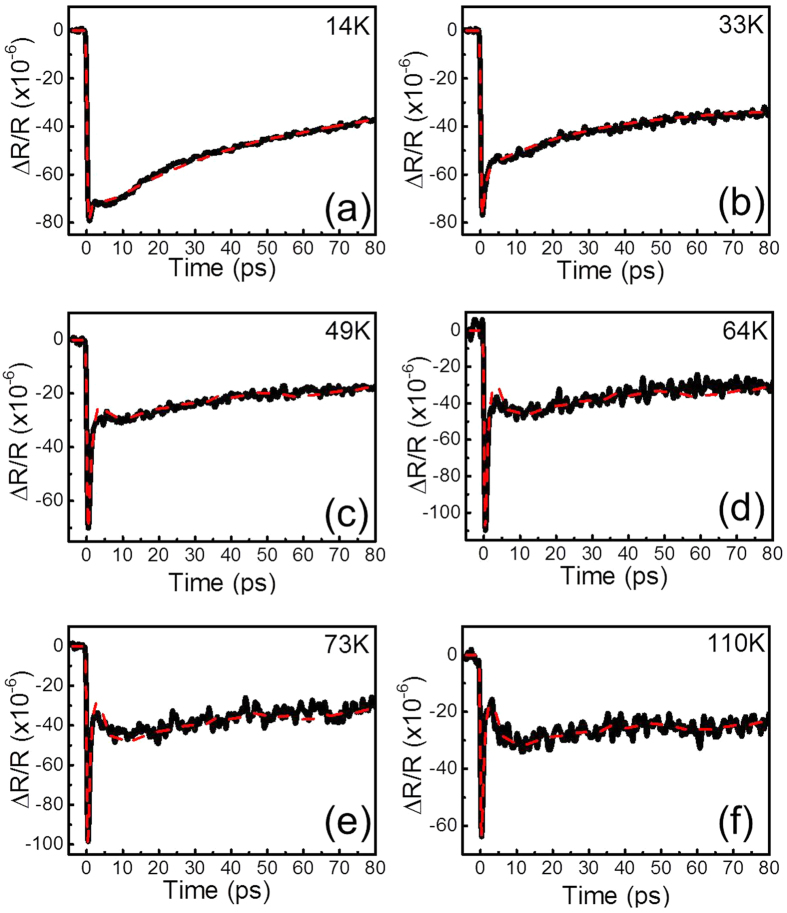
The time-resolved optical reflectivity of BKFA (black solid lines) and the fitting results (red dashed lines) at (**a**) 14 K, (**b**) 33 K, (**c**) 49 K, (**d**) 64 K, (**e**) 73 K, and (**f**) 110 K.

The magnetic susceptibilities of BKFA as a function of temperature were measured, as shown in Fig. 1. The SC temperature has been evaluated as 36 K. We conducted temperature-dependent transient optical reflectivity measurements of BKFA. Detailed experimental conditions can be found in the Methods section. Figure 2 shows the measurements for six representative temperatures. As shown in Fig. 2(f), the 110 K trace shows a fast exponential function with a time constant of < 1 ps, followed by a broad negative hump at ~12 ps. The ultrafast relaxation time within 1 ps should be attributed to thermalization of non-Fermi distribution of electrons^39^.

We ascribe the negative humps at ~12 ps to the effects of propagating coherent longitudinal acoustic phonons along the depth direction. In transparent or semi-transparent media, the propagation of coherent acoustic phonons induces temporal sinusoidal oscillation due to coherent Brillouin scattering^40^. The oscillation period of multiple cycles is experimentally resolvable and is determined by the refractive index, sound velocity, optical wavelength, and incidence angle of the optical probe in the media^40^. However, the feature of transient reflectivity due to propagating phonons in highly absorptive materials, such as the BKFA we studied, is not trivial. In order to understand the acoustic signals, we have used the finite difference time domain method to simulate the temporal evolution of longitudinal strain distributions 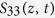 (or longitudinal acoustic phonons) in BKFA^41^,^42^.

The time-resolved optical reflectivity can be represented as^43^


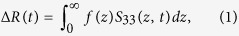


where


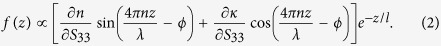


**Figure 3:**
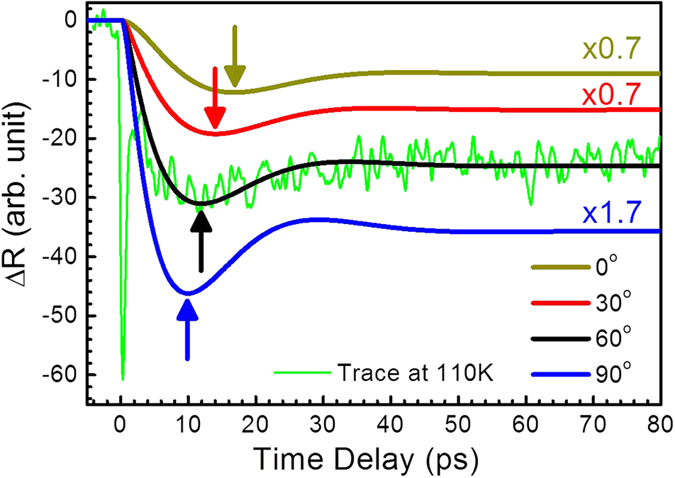
The calculated time-resolved optical reflectivity due to propagating acoustic phonons in BKFA. The center of the negative hump, as shown by arrows, varies for different phases of the photoelastic constant. Note that the traces for some phases are scaled for easier comparison. The green line shows the rescaled trace at 110 K from Fig. 2(f).

*n* and 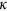 are respectively the real part and the imaginary part of the complex refractive index of the sample, *z* is the depth position from the surface, *λ* is the optical wavelength in vacuum, and *l* is the optical absorption length. *ϕ* is a constant, which is related to *n* and 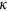
43. 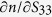 and 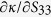 are the real part and the imaginary part of the photoelastic constant, respectively. The photoelastic constant can also be expressed as 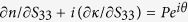. For calculation, we assumed that the initial condition of 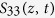 is proportional to exp(−*z*/*l*), where 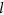 = 27.7 nm at 400 nm^41^. By using the indices of refraction 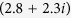 at 800 nm and 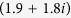 at 400 nm for BKFA^41^, 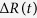 can be calculated following Eq. (1). Figure 3 shows the rescaled 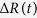 for a few representative phases of photoelastic constants (*θ*). Note that 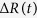 for different *θ* in Fig. 3 can be compared since all of the other parameters, including *P*, are kept the same. The centers of the negative hump vary for different values of *θ*. Therefore, one could not determine the phonon oscillation period simply from the first dip time. According to our fitting results in Fig. 3, the best fit of *θ* was 60° ± 5° for BKFA at 110 K. This is similar to the phase of the photoelastic constant of Cr metal thin film^44^. The amplitude of the photoelastic constant *P* could not be obtained in the present study because the strain under our experimental condition was unknown. Further information such as thermal expansion coefficients and heat capacities should be determined for *P*.

**Figure 4:**
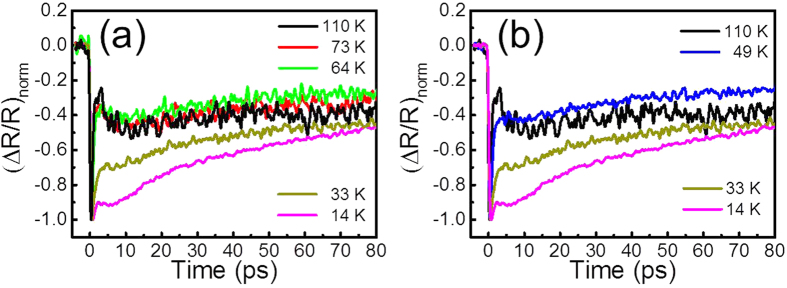
The normalized traces from Fig. 2. (**a**) The traces below T_c_ (36 K), such as those at 14 K and 33 K, clearly show additional relaxation due to the SC gap. (**b**) The 49 K trace still has the SC-gap related relaxation component on the order of several tens of picoseconds.

Figure 4 shows the normalized traces from Fig. 2. Above T_c_ (36 K) in Fig. 4(a), the normalized traces are roughly the same after 10 ps. It should be noted that slower processes such as acoustic phonons, carrier diffusions, and heat diffusions should not be sensitive to the temperature. However, we found additional relaxation components with time constants on the order of several tens of picoseconds in SC state such as at 14 K and 33 K, shown in Fig. 4(a). We attribute these temperature-dependent components to quasiparticle relaxation due to the SC gap^45^.

In order to quantitatively study the temperature-dependence of the carrier dynamics, the transient reflectivity *A*(*t*) can be represented by


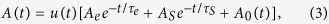


where 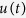 is the step function; 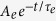 is used to account for the contribution from electron thermalization^39^; 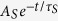 is used to represent the quasiparticle relaxation due to the SC gap; 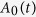 accounts for all of the slower processes such as acoustic phonons, carrier diffusions, and heat diffusions. However, there is no simple analytical form for 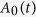. Experimentally, we found the normalized traces above 110 K are almost the same. We assumed that 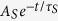 in Eq. (3) vanishes above 110 K. In order to experimentally obtain 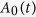, we averaged 5 traces at 130 K, 135 K, 140 K, 145 K, and 150 K. 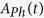, primarily due to acoustic phonons, was thus obtained after further smoothing by adjacent averaging to eliminate the feature of 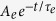. In Eq. (3), 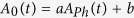 was used for fitting, and 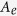, 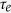, 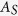, 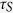, *a*, and *b* are the fitting parameters. *A*(*t*) was convolved with the cross-correlation of pump and probe pulses to quantitatively fit the experimentally recorded 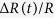 traces below 110 K.

**Figure 5:**
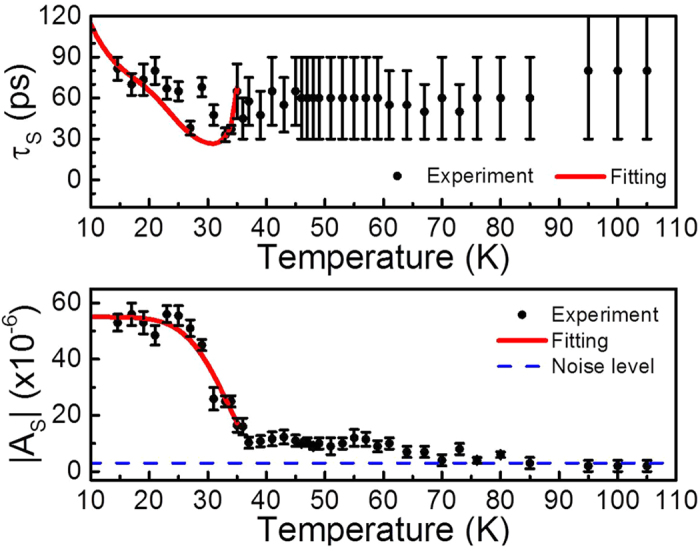
(**a**) *τ*_*S*_ and (**b**) 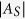 of the SC quasiparticle relaxation term in slightly underdoped BKFA as a function of temperature. The black circles represent the experimental data and the solid red lines represent the fitting curve with Δ(0) = 12 meV. The dashed blue line in (**b**) represents the noise level. The error bars for *τ*_*S*_ are larger above T_c_ due to the relatively small amplitude 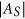.

According to our temperature-dependent analysis, 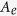 and 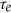 did not show any systematic trend and will not be discussed. Fig. 5(a,b) show the temperature-dependent 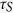 and 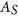. From high temperature to low temperature, 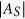 dramatically increases beginning at T_c_ (36 K) and roughly saturates below 25 K. 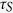 also has a peak at T_c_. These features were similar to those of quasiparticle relaxation due to the SC gap in other iron-based superconductors^26^,^29^,^30^,^46^. Although iron-based superconductors have multiple bands with different SC gaps, a temperature-dependent one-gap system based on the Rothwarf-Taylor (RT) model was still adequate to estimate the SC gap size for FeSe^46^, LiFeAs^26^, Ba_0.6_K_0.4_Fe_2_As_2_ 30, and SmFeAsO_0.8_F_0.2_ 29.

We also used the RT model to interpret and fit our data^45^. The transient temperature near the surface of BKFA exceeds T_c_ after the optical pulses excite the quasiparticles. It thus creates high frequency bosons with energy 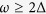 and breaks the Cooper pairs. The relaxation time reflects the population of high frequency bosons and the recovery time of the SC state when the heat escapes from the optically probed depth. In brief, 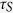 is the recovery time of superconductivity after suppression by photocarriers^45^,^47^.

We assumed a temperature-dependent gap 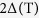, which is proportional to 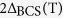 following BCS temperature dependence. 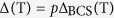, where *P* is the fitting parameter. The numerical solution 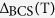 without approximation was used for fitting. 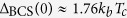, where 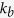 is the Boltzmann constant and T_c_ = 36 K. According to the RT model, the excited population of quasiparticle 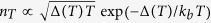. The relations between 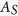, 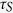 and 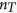 are


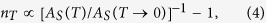


and


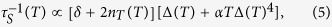


where *δ* and *α* are fitting parameters following the criteria described in ref. 30. The fitting curves were arbitrarily scaled due to the proportionality.

The gap size 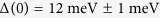 was obtained from the fitting results based on the BCS temperature-dependent one-gap system. The red lines in Fig. 5(a,b) show the fitting curves with 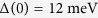. The temperature-dependent 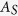 below T_c_ shows good agreement with the RT model, while the temperature-dependent 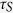 does not agree well. However, 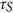 still shows the characteristic trend of the RT model, the “U” shape below T_c_. From low temperature to high temperature, 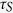 first decreases and then dramatically increases when the temperature approaches T_c_^30^. It should be noted that this is a simplified model. Actually, the transient optical reflectivity should be governed by the contribution of quasiparticle relaxation in all bands due to the multiband feature of iron-based superconductors. According to ARPES measurement results of optimally doped BKFA, Δ is ~12 meV at the inner/outer electron pockets (*γ*/*δ* bands) and the inner hole pocket (*α* band) while Δ is ~6 meV at the outer hole pocket (*β* band)^22^,^23^,^48^,^49^. Our obtained 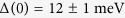 agrees well with that of *α*, *γ*, *δ* bands in BKFA. The uncertainty of the fitting results with the simplified one-gap model might be attributed to different SC gaps of multi-bands in BKFA.

From Fig. 5(b), we have noticed that 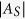 does not vanish above T_c_, which is similar to the phenomenon observed in underdoped BKFA with magnetic transition^30^. The SC-related component of relaxation persists up to at least 70 K in our slightly underdoped BKFA without magnetic transition. Note that the noise level in Fig. 5(b) was obtained by a strict definition of peak-to-peak noise fluctuations in 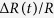, which means the onset temperature could be even higher. It should be noted that the error bars for *τ*_*S*_ are larger above T_c_ due to the relatively small amplitude *A*_*S*_. However, the relaxation component *A*_*S*_ (after consideration of the error bars and noise level) indeed persist from below T_c_ to above T_c_. This phenomenon can also be clearly observed without quantitative analysis. The normalized traces in Fig. 4(b) reflect the percentage of different contributions from 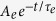, 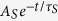, and 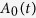 in Eq. (3). For temperature above 110 K, the normalized traces are the same and are only composed of 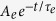 (with 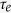 ~1 ps) and 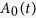. However, for temperature below T_c_ such as 14 K and 33 K, 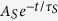 (with *τ*_*S*_ of several tens of ps) is relatively strong and overwhelms some features of 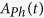 (as shown in Fig. 3 that the signal decreases prior to the minimum at ~12 ps). As shown in Fig. 4(b), these can be clearly observed in the normal state, 49 K trace, which still has the relaxation component 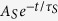. According to our quantitative analysis in Fig. 5(b), the relaxation component due to the SC gap persists at least up to 70 K.

This phenomenon was similar to that found in underdoped BKFA with magnetic transition^30^, and was attributed to the pseudogap (or probably partial gaps) in the normal state. The main difference is that our slightly underdoped samples do not have magnetic transition. In addition, the pseudogap of slightly underdoped BKFA (without magnetic transition) was also found by optical conductivity measurement^31^. Kwon *et al.* found that the gap feature in the range between 50 cm^−1^ and 150 cm^−1^ (corresponding between 6 meV and 18 meV) continuously existed from the SC state to the normal state up to 100 K^31^. Our experimental results and previous reports^30^,^31^ support the proposition that the pseudogap in BKFA should be the precursor of the SC gap because the same SC-related relaxation component in the SC state persists up to the normal state.

It is worthy to note that Chia *et al.* also investigated the optimally doped BKFA and did not observe a significant pseudogap feature above T_c_^30^. We argue that the BKFA sample of our present study should be slightly underdoped since its T_c_ = 36 K is slightly lower than the T_c_ = 37 K of BKFA reported in ref. 30. We also found a similar situation for ARPES measurements in that a pseudogap was found in BKFA with T_c_ = 35 K 22 but was not clearly found in BKFA with T_c_  = 37 K 23. A pseudogap was also clearly observed with optical conductivity measurement in slightly underdoped BKFA without magnetic transition^31^. We thus suggest that the contradictory reports^22^,^23^,^30^,^31^ actually resulted from the slightly different concentrations of K near optimally doped BKFA. Our results and previous reports^22^,^31^ indicate that a pseudogap still exists in slightly underdoped BKFA without magnetic transition. It is interesting that no strong evidence of a pseudogap has been found in optimally doped^30^ or overdoped BKFA, although a shadow gap was predicted recently^50^. Nevertheless, a pseudogap was found in the overdoped regime of other 122 system compounds^28^,^32^,^33^,^34^.

In the 1111^35^,^36^, 111^26^,^37^,^38^, and cuprate^51^,^52^,^53^ systems, it was reported that a pseudogap can coexist with the SC gap. The infrared pseudogap in Co-doped and P-doped BaFe_2_As_2_ (122 systems) was also reported to be unrelated to superconductivity^33^. However, our findings and previous results^30^,^31^ lead us to suggest that the pseudogap in K-doped BaFe_2_As_2_ is the precursor of superconductivity. We suggest that partial gaps along certain momentum might be opened in the normal state and they evolve to complete SC gap below T_c_. Note that the term “pseudogap” was widely used in the literature to specify a gap or a partial gap of which the physical origin is unknown. They may have different physical origins. Our experimental evidence indicates that the origin of the pseudogap in BKFA is different from that of other iron-based superconductors with coexisting pseudogap and SC gap in the SC state^26^,^28^,^32^,^33^,^34^,^35^,^36^,^37^,^38^. We would like to highlight that K atoms are doped out of the FeAs planes while Co and P are doped in the FeAs planes. It was suggested that in-plane doping or out-of plane doping would affect how SC gap forms^54^. A similar situation may occur in the formation of the pseudogap, leading to different features between (BaK)Fe_2_As_2_ (out-of-plane doping systems) and other in-plane systems such as BaFe_2_(CoAs)_2_ and BaFe_2_(AsP)_2_.

## Conclusions

We have utilized ultrafast optical spectroscopy to investigate carrier dynamics in slightly underdoped BKFA without magnetic transition. The signals of acoustic phonons were quantitatively studied. We have also analyzed the quasiparticle relaxation due to the SC gap. Although the doping concentration of BKFA does not have a magnetic transition and is nearly optimally doped, we still found pseudogap feature at least up to 70 K in normal state. Our experimental results support the proposition that the pseudogap in BKFA should be the precursor of the SC gap. We have highlighted the unique pseudogap feature in K-doped BaFe_2_As_2_, which is different from that in other iron-based superconductors.

## Methods

### Experimental Setup

The sample was cleaved to reveal a shining surface, and mounted on the holder of the cryostat in an Ar-filled glove box. After the cryostat was moved out from the glove box, the pressure of the chamber was immediately lowered to below 10^−4^ mtorr to avoid oxidation. Typical pump-probe (400 nm–800 nm) measurements were conducted. The polarization of the pump beam is orthogonal to that of the probe beam. But, the polarization was not intentionally aligned to the crystal axis of the sample. The repetition rate was 8 MHz and the optical pump fluence was 5~10 μJ/cm^2^. The pump was modulated at ~1 MHz with an acousto-optical modulator (AOM). The full width at half maximum (FWHM) of the temporal cross-correlation of the pump and probe pulse was ~400 fs, which determined the temporal resolution. Time constants close to the temporal resolution could still be accurately determined after the deconvolution process. A color filter was placed in front of the photodetector to eliminate pump light leakage. We recorded the reflectivity variation of the probe pulse as a function of time delay.

Under our experimental condition, the traces were in the linear region above 50 K. The normalized traces for optical pump fluence of both 5 μJ/cm^2^ and 10 μJ/cm^2^ did not show significant difference. At the lowest temperature cooled with liquid He, the normalized traces were different for optical pump fluence of 5 μJ/cm^2^ and 10 μJ/cm^2^. The steady-state laser heating still occurred under our experimental condition especially at low temperatures. The real temperature of the sample increased with increasing optical pump fluence. The temperature was calibrated as described in the next section. However, it should be noted that fluence-dependent measurements cannot be used to verify the linearity in the SC state of BKFA. It was reported that the decay rate of the quasiparticles due to the SC gap varies with optical pump fluence^55^. In our temperature-dependent measurements, the optical pump fluence was 5 μJ/cm^2^ below 50 K, which was reasonably low compared with previous experiments on iron-based superconductors^25^,^26^,^29^,^30^,^46^,^55^. For enhancement of the signal to noise ratio, the optical pump fluence was 10 μJ/cm^2^ for measurements above 50 K because they are in the linear region. The traces above 50 K were rescaled for comparison.

### Temperature Calibration

The temperature of the sample was 10 K calibrated from the temperature measured by the thermometer. According to Fig. 2(b) in ref. 55, the features of normalized traces in SC and non-SC states under the same pumping fluence can be easily distinguished. We could thus obtain the temperature difference between the real temperature T_c_ of the sample and the temperature measured by the thermometer.

## Additional Information

**How to cite this article**: Lin, K.-H. *et al.* Ultrafast dynamics of quasiparticles and coherent acoustic phonons in slightly underdoped (BaK)Fe_2_As_2_. *Sci. Rep.* **6**, 25962; doi: 10.1038/srep25962 (2016).
